# Losing Wallets, Retaining Trust? The Relationship Between Ethnic Heterogeneity and Trusting Coethnic and Non-coethnic Neighbours and Non-neighbours to Return a Lost Wallet

**DOI:** 10.1007/s11205-016-1264-y

**Published:** 2016-02-19

**Authors:** J. Tolsma, T. W. G. van der Meer

**Affiliations:** 1 Department of Sociology, Radboud University Nijmegen, PO Box 9104, 6500 HE Nijmegen, The Netherlands; 2 Department of Political Science, University of Amsterdam, Nieuwe Achtergracht 166 (r10.09), 1018 WV Amsterdam, The Netherlands

**Keywords:** Trust, Ethnic heterogeneity, Neighbourhood, Egohood, Wallet items

## Abstract

The constrict claim that ethnic heterogeneity drives down social trust has been empirically tested across the globe. Meta-analyses suggest that neighbourhood ethnic heterogeneity generally undermines ties within the neighbourhood (such as trust in neighbours), but concurrently has an inconsistent or even positive effect on interethnic ties (such as outgroup trust). While the composition of the living environment thus often seems to matter, when and where remain unclear. We contribute to the literature by: (1) scrutinizing the extent to which ethnic heterogeneity drives down trust in coethnic neighbours, non-coethnic neighbours, unknown neighbours and unknown non-neighbours similarly; (2) comparing effects of heterogeneity aggregated to geographical areas that vary in scale and type of boundary; and (3) assessing whether the impact of heterogeneity of the local area depends on the wider geographic context. We test our hypotheses on the Religion in Dutch Society 2011–2012 dataset, supplemented with uniquely detailed GIS-data of Statistics Netherlands. Our dependent variables are four different so-called wallet-items, which we model through spatial and multilevel regression techniques. We demonstrate that both trust in non-coethnic and coethnic neighbours are lower in heterogeneous environments. Trust in people outside the neighbourhood is not affected by local heterogeneity. Measures of heterogeneity aggregated to relatively large scales, such as, administrative municipalities and egohoods with a 4000 m radius, demonstrate the strongest negative relationships with our trust indicators.

## Introduction

In seven years, over 100 studies have investigated the constrict proposition, the claim that ethnically heterogeneous environments undermine pro-social attitudes and behaviours of the residents within them, not only ties between ethnic groups but even ties within ethnic groups. Recent review articles (Portes and Vickstrom 2011; Schaeffer 2014; Van der Meer and Tolsma 2014) have shed some light on the resulting ‘cacophony of empirical findings’. Van der Meer and Tolsma (2014) conclude that ethnic heterogeneity does not consistently erode all aspects of social cohesion.^1^ Especially in countries other than the US, the evidence is rather inconsistent. The unconditional and almost apocalyptic claims that found their way to the general media (Hallberg and Lund 2005) are thus vastly overblown. Yet, while the inconsistent evidence led Portes and Vickstrom (2011) to conclude that the scientific and political fuss is unwarranted, this conclusion may be premature: these meta-studies also illustrate the lack of systematic understanding of the conditions under which ethnically heterogeneous environments affect social cohesion.

It is still unclear which relations are sensitive to ethnic heterogeneity. Indicators of cohesion may or may not be restricted in scope to the neighbourhood (such as trust in neighbours vs. generalized trust) and may or may not be targeted to members of specific ethnic groups (trust in coethnics vs. trust in members of ethnic outgroups). From previous research, there are indications that ties explicitly bound to neighbourhoods are quite consistently negatively related to heterogeneity (Finney and Jivraj 2013; Guest et al. 2008; Koopmans and Schaeffer 2015; Letki 2008; Putnam 2007; Rios et al. 2012; Schaeffer 2013; Twigg et al. 2010; Völker et al. 2007; but see f.i. Mata and Pendakur 2014 for an exception). Concurrently, there is no clear consensus on the direction of the relationship between heterogeneity and indicators of interethnic cohesion. While numerous studies point to negative effects of heterogeneity on interethnic relations—especially outside the constrict proposition literature and when heterogeneity is aggregated to relatively large geograpahic areas (e.g. Quillian 1996; Scheepers et al. 2002)—inter-ethnic relations are also commonly found to be positively related to ethnic heterogeneity of local environments (e.g. Lancee and Dronkers 2011; Tolsma et al. 2009; Vervoort et al. 2011; for overviews see Pettigrew and Tropp 2006; Van der Meer and Tolsma 2014; but see Rudolph and Popp 2010 that demonstrates negative effects of concentration of blacks and Hispanics in US municipalities on interracial trust). Even less is known about the way heterogeneity impacts intra-ethnic relationships, i.e. attitudes towards and relationships with coethnics. This is somewhat surprising as it was especially this part of Putnam’s constrict claim—that both cohesion between *and* within ethnic groups will be eroded by ethnic heterogeneity—that created most of the fuss in the first place. Moreover, what has remained unclear, both theoretically and empirically, is what happens when the scope and target dimensions of cohesion intersect; the extent to which ethnic heterogeneity affects inter-ethnic and intra-ethnic ties within the neighbourhood differently. In the present contribution we will focus on social trust, because it is a core component of social cohesion and we are able to systematically vary the scope and target of trust in our measurement instruments. The first research question we will address is: To what extent does ethnic heterogeneity differently affect (a) trust in neighbours versus trust in non-neighbours and (b) trust in coethnic neighbours versus trust in non-coethnic neighbours?

The inconsistent results in the constrict literature may in part be due to the problem of pinpointing the relevant geographic environment and acknowledging that this relevant residential context may depend on the indicator of cohesion studied. Effects of ethnic heterogeneity on indicators of cohesion are generally rather small in comparison with individual determinants of social cohesion (Guest et al. 2008). This does not mean that neighbourhood heterogeneity does not matter. As Sharkey and Faber (2014) argue, the question “Do Neighbourhoods matter?” is flawed in itself, one of the reasons being that individuals are affected by social processes operating at different scales. Different contexts may affect social trust in different ways (Baybeck 2006). Although this modifiable areal unit problem (MAUP) is a classic problem in statistical analysis of geographical data, most scholars, following Putnam (2007), focused on the effects of heterogeneity aggregated to administratively defined ‘neighbourhoods’. We will not adopt a single definition of neighbourhood but instead will both vary the scale (small to large) and type of boundary (administratively defined vs. defined by distance) in our conceptualization of ‘the neighbourhood’. This brings us to our second research question. *In which geographical area (scale and type of boundary) does ethnic heterogeneity most strongly affect social trust?*

If residential areas are natural entities that shape relevant boundaries and become residents’ frame of reference, heterogeneity effects should be limited to that specific area and residents’ precise location within these areas would not matter. The standard multi-level models in the field indeed assume that spatial error-correlation is restricted to the higher level unit alone. However, the administrative neighbourhood may be a more relevant social environment to those residents who live at the heart of this geographic area than to those who live in the outskirts. Similarly, it is likely that the impact of the local residential area itself depends on the composition of the wider, adjacent geographic context (Baybeck 2006). Our final research questions are: *To what extent does the geographic position of the respondent within the local geographic area moderate heterogeneity effects on social trust? To what extent does the level of ethnic heterogeneity of adjacent areas have an additional effect on social trust?*

We thus build on previous research by: (1) moving from generalized trust items to particularized trust items which we vary systematically on the scope and target dimension; (2) applying different conceptualizations of the neighbourhood; (3) introducing spatial thinking into the heterogeneity-cohesion literature (Logan et al. 2010). We aim to provide more insight into when heterogeneity matters and, thereby, why heterogeneity matters. To answer our research questions we rely on the 2011 wave of the primary dataset ‘SOciaal-Culturele Ontwikkeling in Nederland’ 2011–2012 (‘Religion in Dutch Society’ 2011–2012) or SOCON (Eisinga et al. 2012). SOCON consists of a representative sample of the native Dutch population. We designed ‘wallet items’ to disentangle trust in coethnics from trust in non-coethnics (referring to the target dimension of trust) and trust in neighbours from trust in non-neighbours (referring to the scope dimension of trust). We geocoded the residential address of each respondent and linked these exact latitudes and longitudes to publically available, high resolution GIS data of Statistics Netherlands. This grid cell dataset provides information on characteristics of each 100 by 100 m geographic area (such as demographic composition and housing values) that will be used to construct measures of ethnic heterogeneity and socio-economic status aggregated to egohoods. We also matched our individual-level dataset to publically available datasets of Statistics Netherlands that provide similar information on administrative areas.

## Expectations

### Social Cohesion: From Generalized Social Trust to Trust in Specific Others

While the standard generalized trust question “Generally speaking, would you say that most people can be trusted or that you can’t be too careful in dealing with people?” is commonly used in the literature on the constrict claim (e.g. Tsai et al. 2011; Dinesen and Sønderskov 2015), it suffers from a range of conceptual issues for the purposes of this study (Glaeser et al. 2000; Nannestad 2008; Reeskens 2013). Most notably, it is unclear in whom people place trust, as the item lacks a manifest alter. Glaeser et al. (2000) conclude that generalized trust measures the respondents’ trustworthiness rather than their trusting attitude.

Our study treats social trust as a relational concept along multiple dimensions. This contribution focuses on two of these dimensions: scope and target. *Scope* refers to the social context to which the trust relationship is restricted, such as the workplace, school classes or specific geographic areas. Here we focus specifically on the geographic scope, because empirical evidence seems to suggest that intra-neighbourhood cohesion is more likely to be eroded by heterogeneity than indicators of cohesion with a broader scope (cf. Van der Meer and Tolsma 2014; Koopmans and Schaeffer 2015). *Target* refers to the nature of the (group of) person(s) to which the trust relationship is restricted. These targets may be institutions (e.g. police, governments) or refer to the ascribed or achieved characteristics of persons (e.g. sex, social class). Our focus on the target dimension is motivated by the fact that the ethnicity of the target plays a pivotal role in the constrict literature. The constrict proposition uniquely states that heterogeneity erodes cohesion between *and* within ethnic groups (Putnam 2007: 144, 149).

We are not the first to acknowledge that both the target and scope of trust matters. Yet, the potentially differential effects of ethnic heterogeneity on trust in various groups in different social contexts have not yet been systematically investigated. This contribution starts to fill this lacuna.

There are two types of explanations why specifically the average level of trust placed in neighbours is lower in heterogeneous environments (cf. Öberg et al. 2011). The homophily principle (McPherson et al. 2001) suggests that interpersonal trust is lower between individuals from different ethnic backgrounds. Moreover, in many western countries, (especially non-western) ethnic minorities tend to have lower levels of trust than majority populations. As cohesion is a relational concept, residents of native Dutch origin may be less eager to place trust in neighbours whom they expect not to reciprocate this trust. Because trust in non-coethnics is lower than trust in coethnics ànd because there are more non-coethnics, trust in the ‘average neighbour’ will be lower in ethnically heterogeneous neighbourhoods.^2^ In line with the understanding of social trust as a relation between a respondent (ego) and his/her neighbour (alter), we can hence speak of an alter-composition mechanism. According to the alter-composition mechanism, observed inter-neighbourhood differences in trust are attributable to differences in characteristics of the dyads present in these neighbourhoods, not to a group-level variable such as ethnic heterogeneity; the same dyad will exhibit the same level of trust regardless of the locality in which the respondent and his/her neighbour live in.

The second type of explanation for why trust is lower in heterogeneous environments starts from a true context-effect of ethnic heterogeneity itself. Heterogeneity in spoken languages and cultural norms may induce feelings of anomie, anxiety about the lack of shared institutional norms and moral values with which to comply (Seeman 1959). Residents in diverse, anomic localities may feel deprived of reliable knowledge on how to interact with fellow residents (Merton 1938). As a result, overall levels of contact may decrease, even further undermining familiarity with people in one’s direct surrounding, including coethnics. Or as Öberg et al. put it (2011: 351–352), it becomes more risky to trust others in diverse networks because residents are less inclined to believe that there are community norms and guidance for appropriate behaviour. The anomie mechanism predicts that heterogeneity will erode trust in non-coethnic and coethnic neighbours alike.^3^

Neither of these mechanisms can explain why ethnic heterogeneity is positively related to interethnic trust. For that, we ought to look at contact theory (Allport [1954] 1979) which poses that positive contact experiences undermine negative stereotypes and reduce negative interethnic attitudes. As perceptions of intra-group homogeneity are reduced, demarcations between the ethnic ingroup and outgroup are weakened to give room for the development of interethnic trust. A straightforward interpretation of the contact mechanism suggests that when inter-ethnic contact increases with increasing levels of ethnic heterogeneity (Blau 1977; Martinović 2013; Van der Laan Bouma-Doff 2007), consequently trust in non-coethnics would go up.

As ethnic heterogeneity increases interethnic contact opportunities, it simultaneously decreases intra-ethnic contact opportunities for the majority group. We assume that, especially when non-coethnics make up a large proportion of the neighbourhood, limited opportunities for contact with coethnic neighbours will lead to less actual contact with, less exposure to, and less familiarity with coethnic neighbours. Although it has been convincingly shown that contact with different types of outgroups reduces hostility towards these outgroups (Pettigrew and Tropp 2006), the impact of (reduced) contact with ingroup members has not been investigated. However, as mere exposure to unfamiliar persons (e.g. such as coethnic neighbours) leads to more positive attitudes towards these persons (Bornstein and Craver-Lemley 2004) and, as argued above, less familiarity with specific neighbours will lead to less trust in neighbours, we expect that in more heterogeneous neighbourhoods trust in coethnic neighbours is lower.

Naturally, and as we will demonstrate below, the composition of the local residential area is likely to be related to the composition of adjacent areas. But once we take into account the composition of this wider environment we no longer expect the local area to affect trust in people outside one’s neighbourhood via the alter-composition mechanism. Similarly, the meeting opportunity and contact theory mechanism argue that the level of heterogeneity within a specific area affects the level of trust within this specific area. Following this line of reasoning, we expect that the ethnic composition of the extra-local area affects trust in people who live outside one’s own residential neighbourhood. On the other hand, feelings of anomie may be an encompassing state of mind: the insecurity of how to act need not disappear when one leaves the residential neighbourhood. This would suggest that levels of heterogeneity of the residential area also affect trust in people outside this area. On top of these mechanisms, there may be spill-over effects, where trust in neighbours (a kind of particular social trust) functions as a stepping stone towards more generalized forms of trust (Glanville and Paxton 2007; Newton and Zmerli 2011; Dinesen and Sønderskov 2015).

Our expectations with respect to the relationships between ethnic heterogeneity of the local neighbourhood and different indicators of trust are summarized in Table 1.

**Table 1:** Expected correlation of ethnic heterogeneity of the local neighbourhood with four different indicators of trust according to different theoretical mechanisms

Theoretical mechanism	Trust in coethnic neighbours	Trust in non-coethnic neighbours	Trust in neighbours (ethnicity unspecified)	Trust in non-neighbours (ethnicity unspecified)
Alter-composition	None	None	Negative	Negative^a^/none^b^
Anomie	Negative	Negative	Negative	Negative
Contact	Negative	Positive	None	None

^a^Due to spill-over effects

^b^Without spill-over effects

### Neighbourhood Scale and Type of Boundary

Although scholars have long discussed the relationship between neighbourhoods, communities, and social capital (Forrest and Kearns 2001), the extent to which neighbourhoods may be perceived as communities with socially relevant boundaries remains unclear. We assume that residents of the same neighbourhood are more alike to one another with respect to trust in neighbours than residents of different neighbourhoods. One source for this similarity, or spatial correlation, is the uneven ethnic distribution across these neighbourhoods combined with heterogeneity effects. As the heterogeneity-trust relationship is the focus of the present contribution, we therefore use the strength of the heterogeneity effect on trust as our evaluation criterion for our neighbourhood conceptualization, where we assume that heterogeneity effects are stronger when aggregated to more relevant areas.

To assess the relevant geographic scale at which ethnic heterogeneity effects are strongest, administratively defined geographic areas are not ideal, because administrative units of the same type (e.g. the municipality) vary substantially in shape and size. More fundamentally, a lack of empirical support for the constrict claim may lie in the use of rather arbitrary administrative boundaries (ranging from zipcodes, and census tracts, municipalities, NUTS2 regions within Europe, or countries) (cf. Fotheringham and Wong 1991). Hipp et al. (2012) propose an alternative to these rather arbitrary aggregations. Independent from Hipp and colleagues, Dinesen and Sønderskov (2015) proposed the same approach: defining neighbourhood as egohoods, ego-centered environments with variable radii. Egohoods are indifferent to boundaries of administrative units, have an identical circular shape for each respondent, and may partly overlap others’ egohoods. Consequently, their scale can be varied by increasing the radius, distance from ego, in incremental steps.

#### Scale

While many daily activities (such as visiting neighbours, walking the dog, taking the children to a playground) take place within a pedestrian neighbourhood with an approximately 500 m radius, broader activities such as “church participation, shopping, socializing and high school attendance typically occur within a 4000 m radius” (Hipp and Perrin 2009: 11; cf. Gundelach and Traunmüller 2014). Dinesen and Sønderskov (2015) found significant heterogeneity effects on generalized trust at small levels of analysis (in egohoods with a radius up to 250 m) but not at larger levels of analysis. They conclude that this indicates the relevance of direct exposure to heterogeneity. Our hypothesis is therefore to expect the strongest heterogeneity effects at a small scale: in egohoods with a radius up to 250–500 m. However, Dinesen and Sønderskov (2015) focused on generalized trust and did not investigate the impact of heterogeneity aggregated to egohoods with a radius larger than 2500 m, although people’s everyday mobility may take place in larger spatial areas (Hipp and Perrin 2009; Gundelach and Traunmüller 2014). We will therefore explore the impact of heterogeneity aggregated to egohoods with a radius up to 10,000 m.

In the Netherlands, the geographic scale of administrative neighbourhoods comes close to 500 m radius egohoods. Although their shape and size varies, the median geographic area of Dutch municipalities (5910 ha) comes close to that of 4000 m radius egohoods (5027 ha). As we already noted, the geographic scale of equivalent administrative areas may be highly disparate. For example, the range in area size of administrative neighbourhoods lies between 3 ha and almost 4000 ha. If small is better, then—ceteris paribus—smaller administrative neighbourhoods, districts and municipalities should demonstrate a stronger relationship between heterogeneity and trust than larger administrative areas of the same type.

#### Boundaries

In the neighbourhood effects literature, there appears to be a silent consensus to adopt administratively defined areas. These administrative neighbourhoods and districts often follow natural demarcation lines (canals, main streets) and are relatively homogeneous with respect to build and consequently of constitution. In the Netherlands, some administrative environments (including all administrative municipalities) are political entities. Hence, administrative units are likely to be relevant and recognizable social contexts in the Netherlands.

Nevertheless, there are several reasons why definitions of local environments that rely on administrative or census defined boundaries are not perfectly internally valid, that is, when residents’ perceptions of neighbourhood boundaries do not align with census defined boundaries. First, these boundary definitions changed over time. For example, in the Netherlands, in the period between 1900 and 2010 the number of municipalities declined from 1121 to 431 in 2010 to 403 in 2014, as the national government wishes to reduce the number of political entities at the local level. It is unlikely that the relevant social boundaries for the residents themselves changed accordingly or at the same pace. Second, although boundaries of administrative units often follow natural demarcation lines, this is not always the case and many are easy to cross. Especially for small areas it is unlikely that social ties—even neighbourly ties—are limited to residents of the same census defined ecological unit. Hence, we compare the relevance of areas with administratively defined boundaries to that of the above-mentioned egohoods, which start from the observation that residents see themselves at the centre of their own neighbourhood (Hipp and Boessen 2013) and that individuals may construct her or his own perception of ‘the neighbourhood’. However, the use of distance (as opposed to administrative functions) to define areas can be just as problematic: as ultimately it also relies on externally determined boundaries and how boundaries of neighbourhoods are perceived by residents may vary and be more fluid. As both administrative units and egohoods have their theoretical advantages and disadvantages, we do not have an a priori expectation on which conceptualization is better to pick up effects of ethnic heterogeneity and we therefore adopt an explorative approach.

### Spatial Thinking: No Neighbourhood is an Island

An exclusive focus on ties between residents in single geographic areas may miss the important ties that link to broader environments (Hipp and Boessen 2013; Hipp et al. 2012; Hipp and Perrin 2009). Befriending someone living close by need not by hindered by a mere administrative boundary. Residents who live at the periphery of their neighbourhood of residence and/or close to surrounding neighbourhoods are likely to cross neighbourhood boundaries more often. This may make their neighbourhood of residence less focal and may consequently result in weaker heterogeneity effects of the neighbourhood of residence.

Whenever residents use the amenities of nearby neighbourhoods (e.g. school, shops, etc.), they expose themselves to the ethnic composition of the surrounding environment. In general, people are quite aware of the ethnic composition of their surrounding neighbourhoods (Crowder and South 2008). We thus expect an additional influence of the level of ethnic heterogeneity of areas surrounding the neighbourhood of residence. For trust in non-neighbours, this broader environment may be especially relevant, as both the alter-composition mechanism and the anomy mechanism may be at work. We will thus investigate the impact of ethnic heterogeneity of the immediate neighbourhood and of heterogeneity outside the immediate neighbourhood. Although this has been done before in research that operationalizes neighbourhoods using census tracts in US, this approach has not yet been adopted before in the literature on neighbourhoods and social cohesion, nor when neighbourhoods are conceptualized as egohoods.

## Data and Methods

### Individual-Level Data: Religion in Dutch Society 2011–2012

Information on respondents is obtained from the survey Religion in Dutch Society 2011–2012 (Eisinga et al. 2012). This dataset covers questions that were specifically designed for this study. The target population consists of non-institutionalized people aged 18–70 living in the Netherlands. A two-step sampling procedure was applied to select individuals within households. First a random sample of addresses from the full registry of postal codes was taken. Second, the ‘last birthday rule’ was applied to select the respondent who would be invited to cooperate. The personal interviews (CAPI) were held between September 2011 and February 2012. The net-response rate was 53 % (*N* = 994).

In this study, we selected only those respondents who were born in the Netherlands and of whom both parents and all four grandparents were born in the Netherlands (*N* = 856). Furthermore, since we need to enrich our data at the individual level with GIS-data, we selected only those respondents for whom we were able to determine the exact latitude and longitude of their residence (*N* = 797). While this led to the exclusion of a small part of the original sample—59 native Dutch respondents who filled in the complete questionnaire online—we did not lose any respondents due to our geocoding procedure.

### Dependent Variables

Our four dependent variables are formed by four different so-called wallet-items (cf. Stolle et al. 2008; Gundelach and Freitag 2014; Mata and Pendakur 2014). The wallet-items have several advantages.^4^ First, unlike generalized social trust, the wallet-items treat trust as a relational characteristic with not only a subject (who trusts) but also an object (who is trusted) and a circumstance (to do what). Second, the consistent frame allows us to differentiate the theoretically relevant object, keeping all else constant. The precise wordings of the questions were:

*‘If you lost a wallet or purse that contained valuable items, how likely is it to be returned with the valuables in it, if it was found by…': *

*…a native Dutch resident of your neighbourhood?;*

*…a Moroccan resident of your neighbourhood?;*

*…someone of your neighbourhood you do not know?;*

*…someone outside your neighbourhood you do not know?.*

The answer categories were: (4) ‘very likely’; (3) ‘likely’; (2) ‘unlikely’; (1) ‘very unlikely’. Each of our respondents thus answered each of the four different wallet items.^5^ With our first two wallet items, we do not ask how likely it is that a lost wallet will be returned by a specific member of an ethnic group but how likely it is that it will be returned *if it is found by a specific member of an ethnic group.* In contrast to natural experiments with purposely ‘lost’ wallets or letters, our measures are thus not hindered by the fact that in some neighbourhoods it will be less likely that a member of a specific ethnic group finds the lost item (cf. Koopmans and Veit 2014). The observed impact of ethnic heterogeneity on trust in coethnic neighbours and trust in non-coethnic neighbours will thus reflect a ‘true’ context effect and not merely differences in the alter composition across neighbourhoods. When we do not specify the ethnicity of the person who finds the wallet, we assume that respondents think of their ‘average neighbour’ and heterogeneity effects may hence also be the result of the alter composition mechanism.

Native Dutch generally refer to migrants and their descendents from Morocco as Moroccans, even though most (also) have Dutch citizenship. We adopted the same terminology in our questionnaire. Moroccans constitute the second largest non-western minority group in the Netherlands (2.2 % in 2014), after the Turks (2.4 % in 2014). From previous research we know that native Dutch prefer their ethnic ingroup the most and that inhabitants from Moroccan origin (and other Islamic groups) are least preferred. We thus contrast ethnic heterogeneity effects for the most and least preferred ethnic group. The responses to the wallet items demonstrated a clear hierarchy, in line with the expected ethnic hierarchy (see Table 2). On average, unknown neighbours are considered less trustworthy than Moroccan neighbours and (unknown) people outside the neighbourhood are trusted the least. Mokken scale analysis (Van Schuur 2003) confirms that this trust hierarchy is not only observed at the aggregate level but also within individuals (scalability coefficient *H* is 0.60, *SE* = 0.03).

**Table 2:** Descriptive statistics wallet items (higher scores indicate more trust, *N*_*i*_ = 789)

Wallet item	Mean	SD	Min	Max
Coethnic neighbour	3.02	0.77	1	4
Non-coethnic neighbour	2.63	0.79	1	4
Unknown neighbour	2.51	0.75	1	4
Unknown non-neighbour	2.25	0.74	1	4

### Covariates at the Individual-Level

Our exclusive focus on native Dutch respondents ensures that ego-ethnicity-effects do not interfere with our model. We do include control variables for other individual level determinants of trust. *Gender* with males coded as (1) and females as (0). *Age* is defined by year of birth. *Education* is measured in years. *Labour market position* is coded in three categories: ‘employed’; ‘unemployed’ and ‘non-employed’. The latter category contains students, pensioners, housewives and the disabled. We also control for net *household income.* Respondents could choose between 12 different income ranges. We used the midpoint values and took the natural logarithm to take into account the skewed income distribution. Missing values on this constructed income variable (10 %) were replaced with the grand mean. *Denomination* consists of the categories ‘no denomination’, ‘Catholic’, ‘Protestants’ and ‘other religion’. The two respondents with missing values for this measure were deleted from the sample. A second indicator of religiosity is *church attendance* measured in the following categories; ‘never’, ‘about once per year’, ‘about once per month’, ‘about once per week’ and recoded in times per year. All respondents—including those without a denomination—were asked about their church attendance. *Household composition* was determined based on marital status (single vs. married) and whether respondents had children who did or did not live at home. This resulted in 6 categories: ‘single, no children’, ‘single, no children living at home’, ‘single, children living at home’, ‘couple, no children’, ‘couple, no children living at home’, ‘couple, children living at home’. The six respondents with missing values for this measure were deleted from the sample. Self Rated Health is assessed with a single item ‘In general, how do you rate your health?’ with answer categories (1) ‘excellent’, (2) ‘very good’, (3) ‘good’, (4) ‘fair’, (5) ‘poor’. All continuous covariates were z-standardized. Our working sample consists of 789 individuals.^6^ Descriptive statistics for covariates at the individual-level are summarized in “Appendix 1”.

### Contextual Data: Administrative Units

**Fig. 1:**
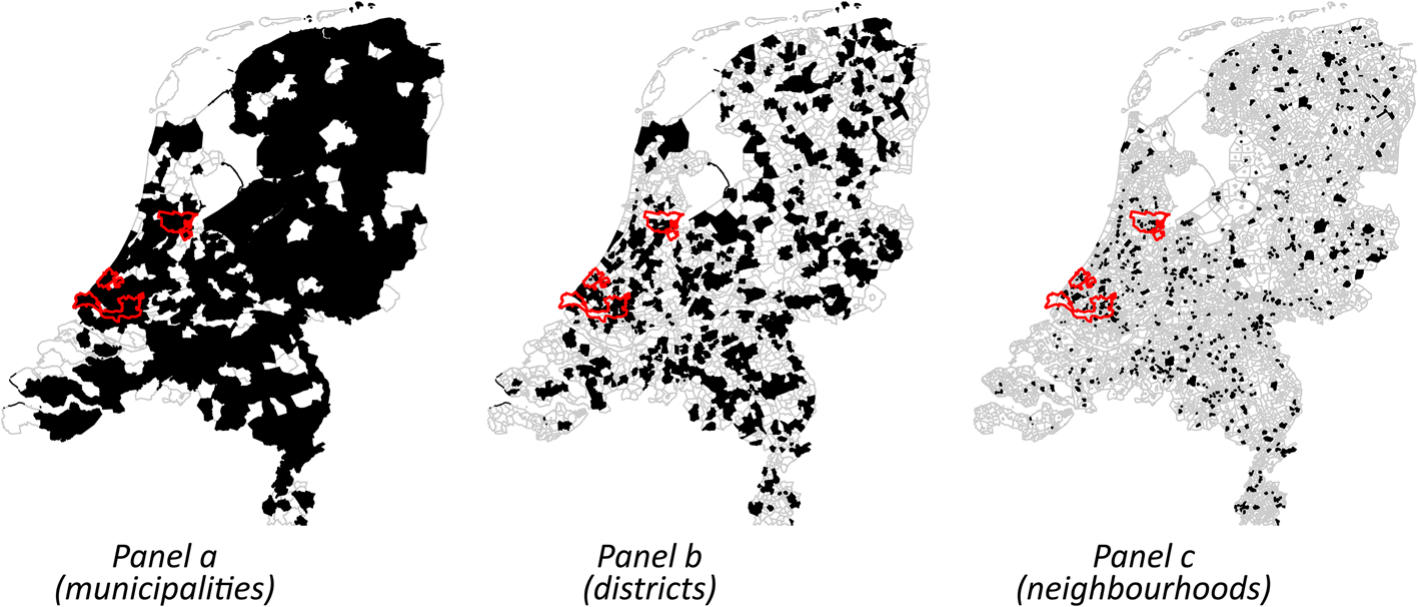
The Netherlands: spatial distribution of the sampled administrative municipalities, districts and neighbourhoods. *Notes*: sampled areas are *black*. Administrative boundaries are *grey*. Municipality boundaries of Amsterdam, The Hague and Rotterdam (from north to south) are *red*. (Color figure online)

Our 789 respondents live in 720 different administrative neighbourhoods (‘buurten’), 579 different administrative districts (‘wijken’) and in 287 different administrative municipalities. The number of respondent suffices with respect to the number of contexts, especially given the sampling methods used in SOCON: we are able to distinguish between individual and contextual effects. Although our dataset at the individual level is relatively small in comparison to previous research, given the spatial distribution of our respondents we have a large sample of higher-level units. This makes our dataset ideal to estimate the impact of characteristics of these contexts. See Fig. 1 for the spatial distribution of the sampled administrative units across the Netherlands. Note that we are not interested to partition variance at the individual- and contextual-level and it is therefore not problematic that we have relatively few respondents per higher level unit (Bell et al. 2008). We use data from Statistics Netherlands to add contextual information to these administrative units.^7^

**Fig. 2:**
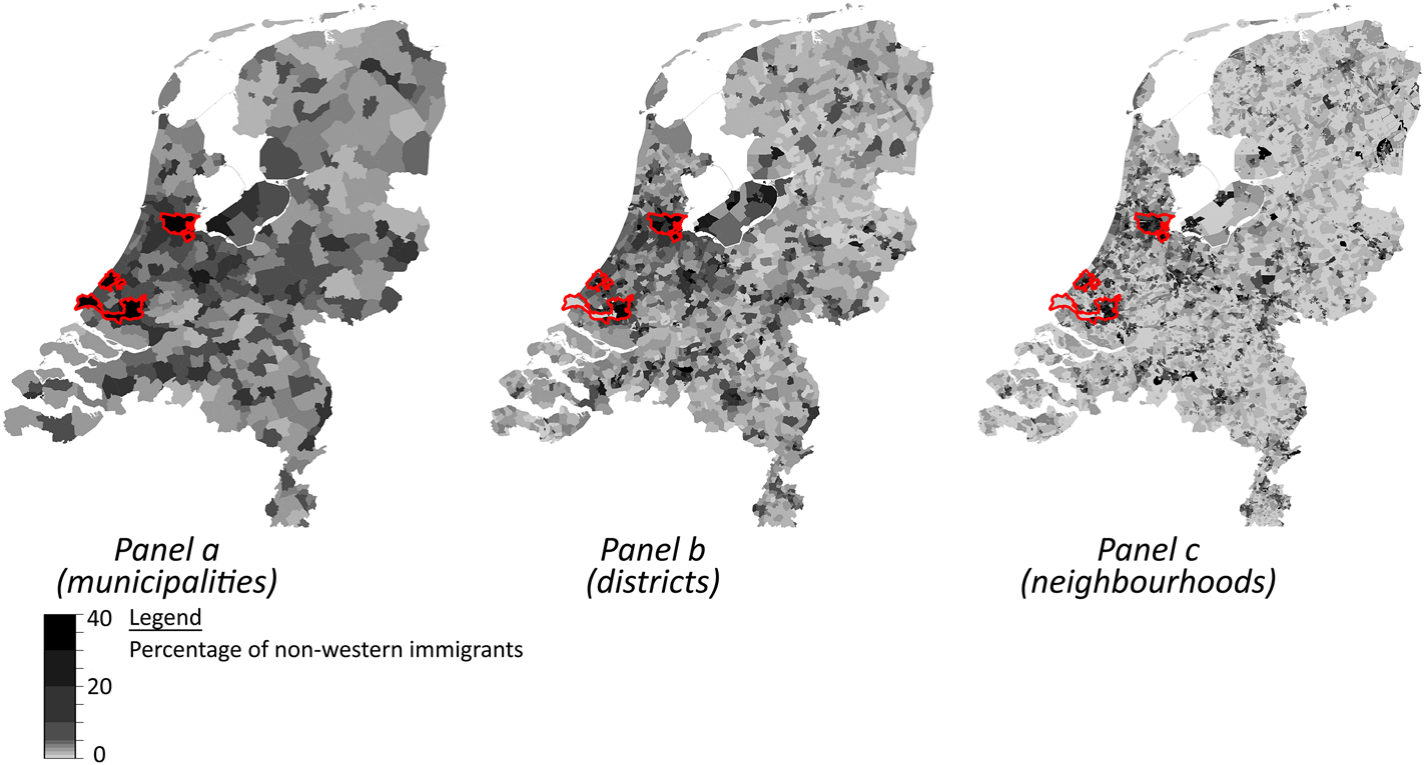
The Netherlands: spatial distribution of non-western minorities in the Netherlands. *Note*s: municipality boundaries of Amsterdam, The Hague and Rotterdam (from north to south) are *red*. (Color figure online)

The ethnic composition of geographic areas, may be characterized in many ways. We operationalize ethnic heterogeneity of the living environments with the measure *migrant stock* (or non-western ethnic density) which refers to the percentage of non-western ethnic minorities, including migrants of first generational status (born abroad) and second generational status (born in the Netherlands or migrated to the Netherlands before the age of six). Our measure excludes western migrants, which constitute approximately 10 % of the population, but an alternative operationalization of migrant stock that also includes western migrants leads to similar outcomes (results available upon request). An ethnic fractionalization, or diversity, measure based on the ethnic categories native Dutch, western ethnic minorities and non-western minorities correlates strongly with our migrant stock measure and, once again, analyses based on this operationalization of ethnic heterogeneity lead to substantially similar results (results available upon request).^8^ Given that our sample only consists of native Dutch respondents and the theoretical shortcomings of diversity measures, we only present the results based on our migrant stock measure. The spatial variation in migrant stock is illustrated in Fig. 2. From panel a it becomes clear that most non-western migrants live in the west of the Netherlands where the largest cities are situated such as Amsterdam, The Hague and Rotterdam. The dark spots in panel b and c are municipalities but as we see there is considerable segregation within municipalities between districts and within districts between neighbourhoods.

To control for the *socio*-*economic status* of the locality we calculated the natural logarithm of the average value of housing units (in Dutch this is called the ‘WOZ-waarde’). Additionally controlling for the percentage of residents with low incomes (incomes below the 40th percentile of the national income distribution) did not lead to substantially different results (results upon request; see also note 16 with respect to additionally controlling for population density). As an indicator of the geographical scale of our administrative units, we used *area size* in square meters. To determine the extent to which residents live in the center of their administrative unit we determined the *distance to the geometric centroid* (in meters) of the respective unit. To construct our indicator for *proximity to other administrative units,* we first determined the mean distance between respondents and the centroid of their residential unit. We subsequently counted the number of centroids of other localities that lie within twice this distance.^9^ To define the extra-local neighbourhood—or surrounding area—we adopt an adjacency approach and is thus defined as the geographic area spanned by the administrative units of the same type that share a border with the residential unit.

### Contextual Data: Egohoods

Contextual characteristics of our egohoods are derived from Statistics Netherlands who provide grid data at a very high resolution, namely on every surface area—or grid cell—of 100 by 100 m. This is called a ‘hectare’ (ha) and is equivalent to almost 12,000 square yards, or almost 2.5 acres.^10^ To define egohoods we identified the grid cells of which the centroids were within specific distances (the radii of the egohoods) of the residential address of each respondent. Our smallest egohoods had a radius of 100 m, the largest egohood a radius of 10,000 m. For egohoods with a radius of 1000 m, we also measured characteristics of the extra-local egohood. The extra-local egohood is a concentric ring—or shell—with a radius in the range between 1000 and 5000 m from the residential address of each respondent.

Due to privacy regulations, the percentages of ‘non-western immigrants’ within each grid cell are only provided as a categorical variable. In order to be able to construct migrant stock measures aggregated to egohoods, we need variables at the interval level. The original categories were recoded as follows: 0 into 0; range <0, 10> into 3.22; range [10, 25> into 15.04; range [25, 45> into 15.04; range [45, 67> into 53.29; range [67, − > into 78.04. These values are not chosen arbitrarily but refer to the mean percentage of non-western migrants of those administrative neighbourhoods that fall within the original categories of the grid cells. To assess the reliability of our recoding scheme, we used the grid cell data to constructed measures of migrant stock aggregated to administrative units. The correlation between the thusly obtained migrant stock measures and the official migrant stock figures of the administrative units as provided by Statistics Netherlands are almost perfect (with Pearson’s correlations of 0.92, 0.97 and 0.99 at the administrative neighbourhood, district and municipality level respectively), thereby showing the reliability of our measures based on aggregations of grid cell information.

To control for the *socio*-*economic status* of the egohood we include the (natural logarithm of the) average value of housing units. As we also know the number of housing units in each area, we are able to aggregate this measure to egohoods as well.

For more information on the construction of egohood measures see, for example, Reardon and O’Sullivan 2004. Descriptive statistics for our contextual variables are summarized in “Appendix 2”.

### Methods

When we assess the impact of migrant stock of administrative units, we assume that spatial error correlation is restricted to the administrative unit under scrutiny and we apply standard two-level linear multilevel models, estimated with the package lme4 in R. When we assess the impact of migrant stock of our egohoods, we estimate linear spatial error models with the package spdep in R and use a row-standardized weight matrix, with distance based neighbours (i.e. the radius of the egohood; see for more information Bivand et al. 2008). With this model we closely follow the logic of standard multilevel models but for non-nested data. All our R-scripts are available upon request.

## Results

**Fig. 3:**
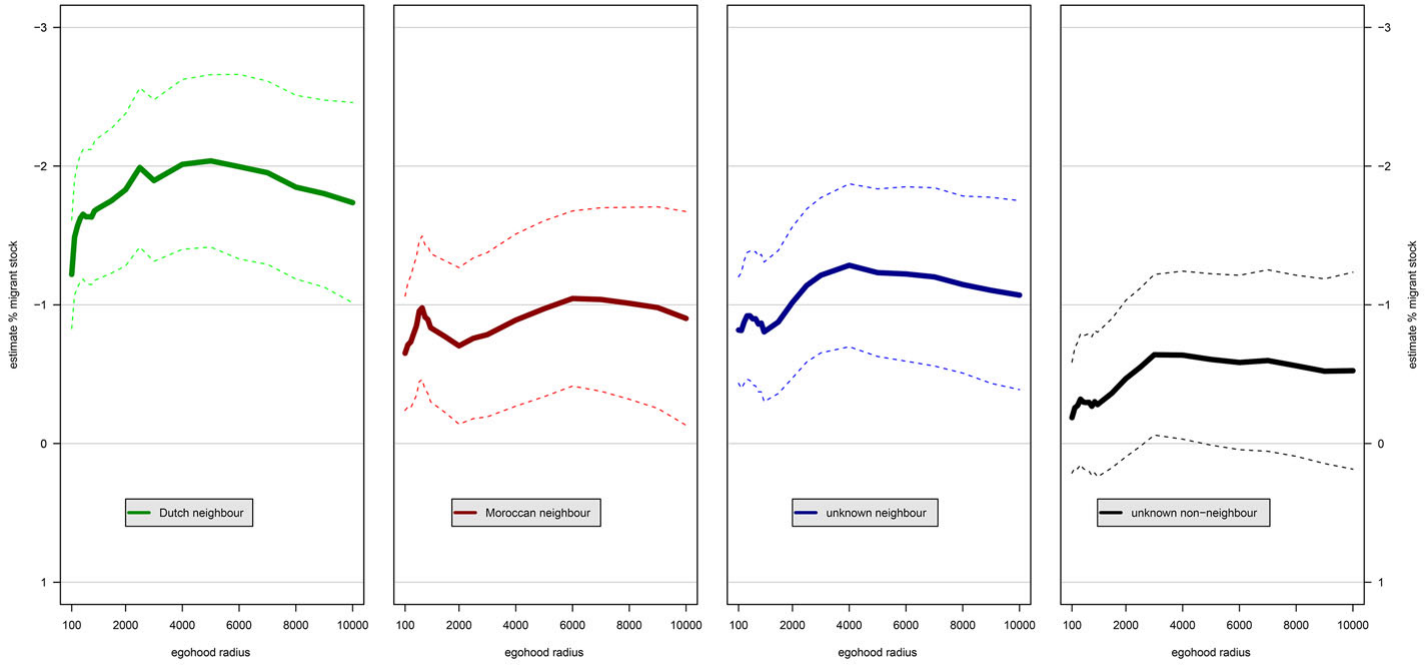
The impact of migrant stock aggregated to egohoods with increasing radii on four different wallet items measuring trust in coethnic and non-coethnic neighbours and trust in unknown neighbours and unknown non-neighbours. Results from linear spatial error models. *Note*: *solid lines* refer to parameter estimate of migrant stock. *Dashed lines* refer to 90 % confidence intervals. (Color figure online)

The results presented below are based on models in which all control variables are included into the explanatory model. The individual-level effects are mostly in line with previous research (see “Appendix 3”, Model 1). Most aspects of trust are higher in more affluent areas (“Appendix 3”, Model 2), with the exception of trust in non-neighbours. The variance at the higher level units (multi-level models) and the labda coefficients (spatial regression models) indicating spatial autocorrelation are relatively small (not shown). This is probably in part because we have few respondents living close to each other.^11^ The impact of migrant stock measured at the level of the administrative neighbourhood, district and municipality is summarized in Table 3, Model 3. The parameter estimates of the effect of migrant stock aggregated to egohoods of different radii, together with the 90 % confidence intervals, are graphically summarized in Fig. 3. To assess the significance of the difference between the estimates of our migrant stock measures between non-nested models (e.g. to test for the difference in heterogeneity effects in contexts of various sizes) we rely on independent-samples *t*-tests.^12^ We also performed three-level multi-level analyses in which the answers to our four wallet items were nested in respondents which were nested in a specific administrative unit. We were then able to directly test whether heterogeneity effects were statistically different for our four trust indicators, given a specific aggregation level of heterogeneity.

**Table 3:** The impact of migrant stock aggregated to different administrative units on four different wallet items measuring trust in coethnic and non-coethnic neighbours and trust in unknown neighbours and unknown non-neighbours

	Administrative neighbourhood				District				Municipality			
	Co-ethnic	Non-coethnic	Un-known nb	Unknown non-nb	Co-ethnic	Non-coethnic	Un-know nb	Unknown non-nb	Co-ethnic	Non-coethnic	Un-known nb	Unknown non-nb
*Model 3*												
Migrant stock	−**1.16**	−**0.72**	−**0.70**	−0.07	−**1.24**	−*0.59*	−**0.76**	−0.26	−**1.86**	−**0.88**	−**1.21**	−*0.63*
*Model 4*												
Migrant stock (ms)	−**1.15**	−*0.62*	−**0.65**	−0.13	−**1.38**	−0.47	−**0.92**	−0.40	−**1.49**	−**0.86**	−**0.96**	−0.25
Area size (as)	0.03	−0.03	0.04	*0.05*	*0.06*	0.01	**0.08**	**0.08**	0.06	0.04	**0.09**	*0.08*
ms × as	−0.07	0.37	0.05	−0.28	−*0.65*	0.24	−*0.77*	−*0.72*	−*0.58*	−0.06	−0.44	−*0.61*
*Model 5*												
Migrant stock (ms)	−**1.18**	−**0.74**	−**0.73**	−0.06	−**1.25**	−*0.54*	−**0.73**	−0.27	−**1.80**	−**1.00**	−**1.21**	−0.44
Distance to centroid (dc)	−0.00	−0.03	−0.01	−0.01	0.04	−0.04	0.03	0.02	**0.10**	−0.00	**0.09**	0.05
ms × dc	−0.21	−0.03	−0.21	0.15	−0.31	0.57	0.03	−0.18	−0.21	0.13	−0.13	−0.27
*Model 6*												
Migrant stock (ms)	−**1.46**	−**0.83**	−**0.76**	−0.15	−**1.75**	−*0.63*	−**0.97**	−*0.64*	−**1.76**	−**0.87**	−**1.07**	−0.51
Proximity to other units (pou)	−**0.08**	0.05	−0.04	−0.02	−**0.09**	*0.07*	−*0.06*	−*0.07*	−**0.12**	−0.03	−**0.10**	−*0.09*
ms × pou	**0.70**	−0.01	0.22	0.19	**0.78**	−0.15	**0.38**	**0.57**	**1.24**	0.35	**0.73**	*0.68*
*Model 7*												
Migrant stock adjacent area	−**1.21**	−*0.60*	−**0.67**	−0.14	−**1.38**	−**0.95**	−**1.09**	−0.54	−**0.84**	−0.12	−0.39	−0.29
*Model 8*												
Migrant stock	−*0.74*	−*0.72*	−0.54	0.06	−0.70	0.07	−0.09	0.19	−**1.78**	−**0.96**	−**1.23**	−0.60
Migrant stock adjacent area	−0.59	−0.00	−0.23	−0.18	−*0.82*	−*1.00*	−**1.02**	−0.69	−0.21	0.22	0.05	−0.08

Results from linear multi-level models

Bold face *p* < 0.05; italics *p* < 0.10 (two-sided)

### Migrant Stock Effects on Different Objects of Trust

First, we discuss to what extent our migrant stock measure affects trust in ‘unknown neighbours’ differently from trust in ‘unknown non-neighbours’. Migrant stock has a significantly stronger negative effect on trust in neighbours than on trust in people outside the neighbourhood. This holds irrespective of our neighbourhood definition. For example, at the neighbourhood level, the parameter estimates for migrant stock are −0.70 (*SE* = −0.27) and −0.07 (*SE* = −0.27), for trust in unknown neighbours and unknown non-neighbours respectively (Table 3, Model 3; t-value of the difference = 3.42). The impact of migrant stock on trust in non-neighbours is even non-significant at the neighbourhood and district level.

Until now it was unclear how to interpret the finding in the literature that especially cohesion within neighbourhoods is negatively related to heterogeneity. The reason for this was because intra-neighbourhood cohesion had almost exclusively been related to measures of heterogeneity aggregated to small scale neighbourhoods. Our results show that the scale of the ecological unit to which heterogeneity measures are aggregated is not the lynchpin, because migrant stock measures aggregated to large environments, such as, municipalities and large egohoods, also negatively and significantly affect trust in neighbours. Instead it really seems to be the scope of the social tie that matters.

Next, we turn to the understudied core of the constrict proposition, that ethnic heterogeneity undermines trust between ethnic groups as well as trust within ethnic groups. Both Table 3 (Model 3) and Fig. 3 show that trust in coethnic neighbours as well as trust in non-coethnic neighbours is lower in environments with larger shares of non-western ethnic minorities. For example, at the municipality level an increase of 10 % points of non-western migrants decreases trust in coethnic neighbours with −0.19 [i.e. 0.1 × −1.86 (*SE* = −0.36)] and trust in non-coethnic neighbours with −0.09 [i.e. 0.1 × −0.88 (*SE* = −0.38)]. Thus, in line with Putnam’s constrict proposition, ethnic heterogeneity deteriorates trust in both ethnic outgroup neighbours *and* ethnic ingroup neighbours.

As stated before, when we explicitly refer to the ethnicity of the target of trust in our measurement of trust, we ‘control for’ the alter-composition mechanism. That we still observe a negative impact of migrant stock on trust in non-coethnic neighbours, or more precisely on Moroccan neighbours, should, hence, be seen as support for the anomie mechanism and implies that ethnic heterogeneity has an impact on top of merely changing the composition of one’s social environment.

The negative effect of migrant stock on trust in native Dutch neighbours is larger than on trust in Moroccan neighbours. The difference is significant at the neighbourhood level (t-value = 2.78), the district-level (t-value = 3.55) and the municipality-level (t-value = 3.65). This is also clearly illustrated in Fig. 3 in which the green line, referring to the impact of migrant stock on coethnic neighbours (i.e. Dutch neighbours), consistently lies above the red line, referring to the impact of migrant stock on non-coethnic neighbours (i.e. Moroccan neighbours). Although the 90 % confidence intervals overlap, the pattern is very consistent and, even according to conservative independent-samples *t*-tests, the differences in effects are significant when heterogeneity is aggregated to egohoods with radii in the range 200–400 and 1500–5000 m. This fits our rationale that negative effects of heterogeneity on inter-ethnic trust may be offset by increased inter-ethnic contact opportunities (cf. Schlueter and Scheepers 2010 for a similar argument; see Koopmans and Veit 2014 for contrary findings), whereas the negative effects of heterogeneity on intra-ethnic trust cannot be similarly offset among the native majority and may be even catalyzed by decreased intra-ethnic contact opportunities. Thus both the anomie and the contact mechanism are probably at work.

We would like to point out that the impact of migrant stock is quite substantial. The impact of an increase of 10 % points non-western migrants in one’s neighbourhood on trust in (non-)coethnic neighbours (0.1 × −1.16(*SE* = −0.27) = −0.12 and 0.1 × −0.72(*SE* = −0.28) = −0.07 when aggregated to neighbourhoods, for trust in coethnic neighbours and trust in non-coethnic neighbours, respectively; Table 3, Model 3) is in the same order of magnitude as the impact of a reduction in self rated health by 1 standard deviation or as one additional year of education (“Appendix 3”).

### Neighbourhood Scale and Type of Boundary

Many authors assumed that heterogeneity effects should be most apparent in smaller geographic contexts. The recent study of Dinesen and Sønderskov (2015) were the first to provide empirical ground for this assumption as these scholars found significant heterogeneity effects on generalized trust at small levels of analysis but not at larger levels of analysis. We hence expected more pronounced heterogeneity effects at smaller scales. We did not find support for this ‘small-is-relevant’ hypothesis. Nevertheless, the relevant scale is very consistent across the used trust indicators. Of the three administrative units in our analysis, it is the ethnic composition of the largest unit, the municipality level, that most strongly affects whether residents expect that a lost wallet with valuables will be returned, even if the wallet is found by a neighbour, but the difference in effect sizes across administrative units are not significant, according to independent-samples *t*-tests.

We basically find the same picture when we turn to the results referring to egohood heterogeneity. Figure 3 shows that the strongest effects are found within egohoods of a radius of 5000 (*b* = −2.04, *SE* = −0.39), 6000 (*b* = −1.05, *SE* = −0.39), 4000 (*b* = −1.28, *SE* = −0.37) and 3000 (*b* = –0.64, *SE* = −0.36) meter for trust in Dutch neighbours, Moroccan neighbours, unknown neighbours and unknown non-neighbours respectively. These radii are in the same order of magnitude as the mean distance of residents to the centroid of their municipality (i.e. 3355.5 m). This proves to be a relevant geographic scale for the formation of trust in the Netherlands like in the US (Hipp and Perrin 2009), regardless of the scope of trust and the target of trust. We do find some indications that the area within which most daily activities take place (a 500 m radius) is more relevant than even smaller and somewhat larger areas; trust in Moroccan neighbours and unknown neighbours show local maxima in effect size at radii of 700 and 500 m, respectively (Fig. 3). This differs from the conclusions by Dinesen and Sønderskov (2015) on Denmark, who found that ethnic diversity aggregated to egohoods with a 80 m radius exerts the strongest negative effect on generalized trust. However, differences in effect sizes across different egohood scales do not reach significance.

Although egohoods and administrative units have radically differently defined boundaries, effect sizes of our migrant stock measures do not differ that much; the effect sizes of migrant stock measures aggregated to egohoods are somewhat larger than the impact of migrant stock measured at the administrative municipality level, but not very substantially so.

### Neighbourhood Space

We expected negative estimates for the interaction of migrant stock with (a) area size and (b) the distance between the residential address of our respondents and the centroid of their locality. We already saw that our starting premise—smaller environments matter more—is not valid. It does not come as a surprise that the impact of migrant stock aggregated to administrative units is also not significantly smaller for respondents who live in larger units (of the same type) (Table 3, Model 4), not even for respondents who live further away from the centre of their unit (Table 3, Model 5).

This does not mean that the spatial location in the locality does not matter. Residents who live relatively close to other localities are less influenced by the level of migrant stock in their official residential unit (Table 3, Model 6); the parameter estimates referring to the interaction ‘migrant stock × number of centroids close by’ are fairly consistently positive and reach significance in 7 out of 12 models.^13^ To investigate this further we turn to the impact of ethnic heterogeneity measures of adjacent areas next.

The Pearson correlation between the respective migrant stock pairs of the residential unit and the neighbouring area are 0.79 and 0.75 for the administrative neighbourhood and district level respectively. There is more variation in the ethnic composition if we compare the surrounding area of large units like the municipality (*r* = 0.29). In Model 7, Table 3 we include our migrant stock measure of the adjacent area into our explanatory model but leave the migrant stock of the residential area out of it. In Model 8 (Table 3), both measures are included simultaneously. The estimated impact of the level of migrant stock of the adjacent area is in the expected direction (Model 7) and, at the neighbourhood and district level the estimated coefficients are even larger than of migrant stock of the residential area (Model 3). However, when both measures are included simultaneously (Model 8), the impact of the adjacent area is no longer significant at the neighbourhood level and, at the district level, the original migrant stock measure is no longer significant. This may be due to the relatively high correlation between the two variables. At the municipality level, we do not observe that the migrant stock of the adjacent area has an additional impact on trust.

Egohoods allow a more flexible operationalization of surrounding areas. We set egohoods with a 1000 m radius as the local environment (as this egohood encompasses the first local maximum), and a shell between 1000 and 5000 m as the neighbouring environment (as this covers the radius with the maximum impact of migrant stock). The Pearson correlation between these two migrant stock measures is 0.62. The parameter estimates referring to the migrant stock in the surrounding area (the ‘shell’), are in the expected direction, significant, and very similar in size as the original migrant stock measure (Table 4, Model 7). When both measures are included simultaneously (Table 4, Model 8) the estimates no longer significantly deviate from null, with the exception of the effect of migrant stock on coethnic neighbours.

**Table 4:** The impact of migrant stock on trust, egohood1000 and its shell

	egohood1000			
	Coethnic	Non-coethnic	Unknown neighbour	Unknown non-neighbour
*Model 3*				
Migrant stock	−**1.59**	−**0.78**	−**0.74**	−0.17
*Model 7*				
Migrant stock shell	−**1.46**	−**0.84**	−*0.73*	−0.22
*Model 8*				
Migrant stock	−**1.28**	−0.53	−0.56	−0.08
Migrant stock shell	−0.60	−0.48	−0.34	−0.17

Bold face *p* < 0.05; italics *p* < 0.10 (two-sided)

All in all we at best find weak indications that the level of migrant stock of adjacent, or neighbouring, areas has an additional impact on top of the impact of migrant stock aggregated to local contexts. That for respondent who live close to other localities migrant stock levels of the local context matter less must be due to other reasons. We come back to this below.

## Discussion and Conclusion

In the face of increasing ethnic heterogeneity and migration, the constrict claim raised concerns across the west. By now it has become clear; however, that ethnic heterogeneity does not consistently undermine all aspects of social cohesion but that eroding effects of heterogeneity exist primarily on intra-neighbourhood cohesion (Van der Meer and Tolsma 2014).^14^ In line with this pattern, we demonstrated that negative effects of heterogeneity on trust are limited to trust in neighbours; trust in neighbours is negatively related to migrant stock, trust in non-neighbours is not.

The crucial innovation of the constrict claim is its emphasis that heterogeneity would reduce both out-group *and* in-group solidarity (Putnam 2007). Surprisingly, effects on in-group trust had hardly been studied to date and effects of ethnic heterogeneity on general attitudes towards, and contacts with, ethnic outgroups oftentimes turned out to be positive rather than negative—at least in field studying the relationship between ethnic heterogeneity and (indicators of) cohesion. In our study, we find both a negative effect of ethnic heterogeneity on trust in coethnic neighbours *and* trust in non-coethnic neighbours.

Most studies in this field investigated heterogeneity effects with measures of heterogeneity aggregated to administratively defined areas. Commonly, the smallest administrative units are assumed to be the most relevant residential environment (e.g. Tolsma et al. 2009; but see e.g. Gundelach and Traunmüller 2014). We tested the hypothesis that the impact of heterogeneity is more pronounced at smaller scales and furthermore recognized that administrative units are just one way to conceptualize ‘neighbourhoods’ (Fotheringham and Wong 1991) that we apply next to egohoods (Hipp and Boessen 2013; Dinesen and Sønderskov 2015). We located the strongest negative effect of ethnic heterogeneity on trust, not to small geographic areas, but rather to relatively large ones: administrative municipalities and egohoods with a 4000 m radius. Effects of ethnic heterogeneity aggregated to egohoods are somewhat larger than effects of heterogeneity aggregated to administrative units. These findings were very consistent but differences in effect sizes across different scales were not very substantial nor reached significance. Apparently, in the Netherlands, among native Dutch and with respect to trusting someone to return a lost wallet, it does not matter that much to which scale heterogeneity measures are aggregated. Unfortunately, we were not able to assess the impact of egohoods with radii in the range between 10 and 100 m. Thus, our result not necessarily contradict the finding of Dinesen and Sønderskov (2015) for Denmark that with respect to generalized trust especially the very local context matters but given the trends in effect sizes reported in Fig. 3, we doubt the same holds true in the Dutch context.^15^ These findings thus call for further research.

We find somewhat stronger heterogeneity effects within egohoods than within administrative units but there is still much room for improvement in defining neighbourhoods. For example, future definitions of neighbourhoods could incorporate distance defined boundaries *and* physical boundaries like roads and rivers, thereby constructing ecological egohoods or ‘eco-egohoods’. Moreover, spatial measures of ethnic heterogeneity with theoretically motivated distance decay functions (you are influenced less by people further away) may be even better to pick up negative effects of heterogeneity on cohesion (cf. Hipp et al. 2012; Reardon and O’Sullivan 2004) than the traditional aspatial measures.

To answer our third research question we investigated whether the strength of the effect of measures of heterogeneity aggregated to administrative units are moderated by where residents live in this geographic area. Living close to other administrative units weakens the impact of the level of heterogeneity of the own residential unit. Yet, surprisingly, the answer to our fourth and related research question was that the ethnic composition of surrounding areas does not offer a substantial additional explanation of trust in one’s neighbours.

Our findings but also the shortcomings of this contribution provide some theoretically promising pathways. Our results rule out that the alter-composition mechanism is the sole, or even most important, factor responsible for lower levels of trust in neighbours in heterogeneous environments. Rather, a combination of the anomie mechanism and the contact mechanism is likely to explain the variation (and lack thereof) in the outcomes. A direct test of the anomie-mechanism is called for. Cross-sectional analyses, such as ours, cannot control for selective residential mobility directly and thus probably underestimate the negative impact of ethnic heterogeneity. Concurrently, we—like most of the broader constrict literature—analyze the effects of static measures of migrant stock. Dynamic measures of migrant stock (percentage change in a specific time period) might be more likely to induce feelings of anomie. More rigorous tests of the relationships between ethnic heterogeneity, anomie and trust would rely on a dynamic perspective, acknowledging moving histories and changing environments.

Small administrative areas are oftentimes more densely populated and respondents who live relatively close to other administrative areas are more likely to live in an urban environment. As both the scale of one’s neighbourhood and its population density are likely to affect contact opportunities, disentangling scale effects from population density effects will shed more light on how contact (and exposure) mediates the relationship between heterogeneity and trust. This, however, will be no easy feat as more densely populated areas—Western parts of the Netherlands and cities—will generally harbor relatively more ethnic minorities (Fig. 2).^16^ Next to population density, income inequality, crime rates, politicization of immigration related issues, and residential mobility rates are all characteristics of one’s neighbourhood, to name but a few, likely to affect feelings of trust. As many of these neighbourhood characteristics are affected by increasing levels of heterogeneity, they will mediate the impact of heterogeneity. Because we did not want to run the risk of ‘over controlling’, we therefore decided not to include these characteristics into our explanatory framework. Naturally, it would be interesting to see to what extent these neighbourhood characteristics explain the link between heterogeneity and contact/anomie, and subsequently trust, but that was beyond the scope of the present contribution.

Once we step away from the more apocalyptic claims surrounding the heterogeneity-cohesion literature, there are some promising inroads to be made to understand the ‘restricted constrict thesis’, that is, why cohesion between and within ethnic groups *in neighbourhoods* is eroded by ethnic heterogeneity. Yet, these inroads require detailed measures of social cohesion, proper definitions of neighbourhoods and heterogeneity and direct tests of the presumed underlying mechanisms.

## Appendix 1

See Table 5.

**Table 5:** Descriptive statistics covariates at the individual-level

	Mean (%)	SD	Min	Max
Gender (male = 1)	0.44	0.50	0.00	1.00
Age	45.88	13.21	18.00	70.00
Education	10.92	3.22	6.00	16.50
*Labour market position*				
Employed	72.24		0.00	1.00
Unemployed	2.53		0.00	1.00
Nonemployed	25.22		0.00	1.00
(Logged) household income	7.86	0.60	5.01	9.21
Missing value household income	0.10	0.30	0.00	1.00
*Denomination*				
No denomination	64.89		0.00	1.00
Catholic	16.35		0.00	1.00
Protestants	14.96		0.00	1.00
Other	3.80		0.00	1.00
*Church attendance*				
About once a week	11.03		0.00	1.00
About once a month	6.84		0.00	1.00
About once a year	25.73		0.00	1.00
Never	56.40		0.00	1.00
*Household composition*				
Single, no children	23.32		0.00	1.00
Single, no children at home	7.22		0.00	1.00
Single, children living at home	12.04		0.00	1.00
Couple, no children	5.32		0.00	1.00
Couple, no children living at home	17.36		0.00	1.00
Couple, children living at home	34.73		0.00	1.00
Self rated health	2.52	1.03	1.00	5.00

## Appendix 2

See Table 6.

**Table 6:** Descriptive statistics contextual level

	N^a^	Mean	SD	Min	Max
*Administrative neighbourhood*					
Migrant stock (in %)	720	8.47	11.17	0.00	85.00
Socio-economic status (logged WOZ)	720	5.46	0.33	4.55	6.65
Area size (ha)	720	185.12	328.63	3.00	3961.00
Distance to centroid (m)	720	419.74	347.76	21.04	3806.28
Proximity to other units (number of centroids in range)	720	3.03	2.72	0.00	22.00
Migrant stock adjacent areas	720	8.71	9.57	0.00	66.50
*Administrative district*					
Migrant stock	579	8.35	9.82	0.00	85.00
Socio-economic status	579	5.48	0.29	4.70	6.45
Area size	579	1645.92	2133.24	3.00	25,272.00
Distance to centroid	579	1176.87	912.54	31.68	7916.44
Proximity to other units	579	3.10	3.13	0.00	18.00
Migrant stock adjacent areas	579	8.73	8.27	0.00	58.18
*Administrative municipality*					
Migrant stock	287	6.21	5.68	1.00	37.00
Socio-economic status	287	5.52	0.24	4.94	6.45
Area size	287	10,431.30	9979.21	696.00	76,539.00
Distance to centroid	287	3088.40	1896.99	123.30	11,644.93
Proximity to other units	287	2.47	1.56	0.00	10.00
Migrant stock adjacent areas	287	8.75	7.76	1.00	34.42
*egohood500*					
Migrant stock	780	8.14	9.89	0	68.28
Socio-economic status	780	5.4	0.31	4.59	6.77
*egohood1000*					
Migrant stock	787	8.37	9.35	0	64.96
Socio-economic status	787	5.41	0.28	4.64	6.48
Migrant stock shell	789	8.15	7.53	0	45.22
*egohood4000*					
Migrant stock	789	8.85	7.75	0	47.17
Socio-economic status	789	5.41	0.21	4.9	6.09

^a^Some residents live in a very rural area (e.g. at farms) this means that there is no statistical information available for small egohoods; some small scale egohoods only encompass grid cells with none or a few residents. Therefore, the number of observations is for small egohoods smaller than 789 (e.g. the number of respondents)

## Appendix 3

See Table 7.

**Table 7:** The impact of individual level characteristics and of mean housing values on four different wallet items measuring trust in coethnic and non-coethnic neighbours and trust in unknown neighbours and unknown non-neighbours

	Coethnic	Non-coethnic	Unknown neighbour	Unknown non-neihbour
	b	b	b	b
*Model 1*				
Intercept	**2.96**	**2.69**	**2.55**	**2.29**
Gender (female = ref.)	−0.01	−**0.11**	−0.07	−*0.12*
Age	0.01	0.07	0.06	*0.06*
Education	0.05	**0.12**	**0.11**	**0.09**
Income	0.04	0.02	0.04	0.02
Missing income (not missing = ref)	0.12	0.06	0.11	0.08
Employment stats (employed = ref.)				
Unemployed	0.02	0.27	0.12	0.14
Nonemployed	−0.11	−*0.14*	−*0.12*	−0.07
Health	−**0.08**	−**0.09**	−0.04	−0.03
Denomination (no denomination = ref.)				
Catholics	**0.20**	0.06	*0.19*	*0.13*
Protestants	0.03	−0.03	−0.02	−0.11
Other denomination	−0.02	−0.06	0.02	−0.08
Church attendance	**0.10**	0.05	0.04	**0.09**
Household composition (single without children = ref.)				
Single with children	0.04	−0.06	−*0.23*	−0.16
Single with children living at home	0.02	0.00	0.07	0.11
Couple without children	−0.14	−0.14	−0.09	−0.01
Couple with children	0.06	0.06	−0.04	−0.02
Coup with children living at home	0.10	0.02	0.01	0.04
*Model 2: Model 1* + *housing values, with cluster correction* ^a^				
Model 2a (administrative neighbourhood-level)				
Housing values	**0.59**	**0.36**	**0.35**	0.09
Model 2b (administrative district-level)				
Housing values	**0.53**	**0.24**	**0.26**	−0.01
Model 2c (adminstrative municipality-level)				
Housing values	**0.44**	*0.24*	**0.25**	−0.02
Model 2d (egohood500)				
Housing values	**0.60**	**0.37**	**0.34**	0.05
Model 2e (egohood1000)				
Housing values	**0.59**	**0.30**	**0.31**	−0.02
Model 2f (egohood4000)				
Housing values	**0.50**	**0.30**	*0.22*	−0.06

Bold face *p* < 0.05; italics *p* < 0.10 (two-sided)

^a^Linear multi-level regression models or linear spatial error models

## Footnotes

1 Ethnic heterogeneity is proposed as a label to encapsulate different indicators of the ethnic composition within geographic areas such as migrant stock (or ethnic density), diversity and segregation.

2 Or phrased otherwise: the mean level of trust in neighbours will be lower.

3 Many authors have observed that ethnic outgroup size aggregated to countries and large geographic areas are related to ethnic threat and ethnic hostility, in line with predictions derived from conflict theory and ethnic competition theory (Bobo and Hutchings 1996; Quillian 1995; Scheepers et al. 2002). When ethnic heterogeneity is aggregated to local living environments, evidence for a positive relation between outgroup size and ethnic threat, and consequently, ethnic hostility, is weaker (Tolsma et al. 2008; Van der Meer and Tolsma 2014; Wagner et al. 2006). We therefore do not discuss the group threat mechanism in the main manuscript although the prediction would be that with increasing outgroup sizes, trust in non-coethnic neighbours would go down and trust in coethnic neighbours would increase.

4 The wallet instrument is not without flaws. Answers are bound to be affected by respondents’ beliefs on the socio-economic status of the finder, which is not unrelated to their ethnicity. To tease out to what extent answers on these wallet items are driven by estimations of the richness of the finder (and the implicitly expected trustworthiness of income groups), a future wallet instrument could include items for ‘rich neighbours’ and ‘poor neighbours’.

5 The wallet items were were not randomized in SOCON. In the second wave of the NEtherlands Longitudinal Lifecourse Study (NELLS; Tolsma et al. 2014), a dataset that became publically available only recently, the same wallet items were included but the order in which they were presented was random for each respondent. Here, the specific sequence did not affect the estimates of heterogeneity.

6 Additional analyses demonstrated that alternative strategies to deal with missing values (i.e. income as categorical variable with a category for ‘missing’ and multiple imputation with the R package mice) lead to nearly identical results. Given the number of models we need to run (366) we opted to describe results on the strategy as described in the main text.

7 Note: More precisely, we use the file: ‘2010-buurtkaart-shape-versie-3.zip’. Retrieved at: http://www.cbs.nl/nl-NL/menu/themas/dossiers/nederland-regionaal/publicaties/geografische-data/archief/2012/2012-wijk-en-buurtkaart-2011-art.htm. Date: 27-03-2013.

8 Ethnic fractionalization is defined as: $1 - \sum\nolimits_{i} {p_{i}^{2} }$, where *p*_*i*_ is the proportion of the respective distinguished ethnic group within the locale. The Pearson correlation between migrant stock and ethnic fractionalization is 0.83, 0.89 and 0.91 at the administrative neighbourhood level, district level and municipality level respectively.

9 This cut-off distance is of course quite arbitrary but different cut-off values—in the same order of magnitude—lead to similar results.

10 Retrieved at: http://www.cbs.nl/nl-NL/menu/themas/dossiers/nederland-regionaal/links/2012-kaart-vierkanten-el.htm. Date: 27-03-2013.

11 This will have little effect on the estimates of our parameters (Bell et al. 2008).

12 We used the T-statistic: T = (A − B)/SE(A − B), where SE(A − B) = sqrt(Var(A) + Var(B) − 2Cov(A, B)). Since models are not nested we do not know Cov(A, B) but assuming zero or positive covariance we know the range of Cov(A,B) : [0, sd(A) × sd(B)]. A conservative test of the significance of the difference would assume independence (i.e. Cov(A, B) = 0). Because even with this conservative test we are able to demonstrate that effects are significantly different, this is what we used.

13 A reviewer pointed out that this finding might reflect measurement error. One could argue that because residents see themselves as living in the centre of their neighbourhood, measures of heterogeneity aggregated to administrative units are not perfectly internally valid, especially for respondents living close to adjacent administrative areas. This is why we also estimated effects of heterogeneity measures aggregated to egohoods. As we do not see substantial differences in effect sizes between egohoods and administrative units of approximately the same scale, we do not think that measurement issues are driving these results.

14 This does not suggest that there are no studies that found evidence on other indicators (see a.o. Gustavsson and Johrdahl 2008; Dinesen and Sønderskov 2015 on generalized social trust); yet, evidence is less consistent on those indicators.

15 Note that also in Denmark a negative impact of heterogeneity aggregated to municipalities on generalized trust is observed (Dinesen and Sønderskov 2012).

16 The correlations between population density measured as the number of residents logged per square kilometer and our migrant stock measure are 0.43, 0.58 and 0.72 at the neighbourhood, district and municipality level respectively. Additional analyses indeed showed that in more densely populated areas trust is lower. Additionally controlling for population density shrinks our migrant stock estimates, especially at the level of larger geographic areas (results available upon request). That said, also when population density is included into the explanatory model, we come to the conclusion that heterogeneity effects are stronger for trust in coethnic neighbours than trust in non-coethnic neighbours, that effects are stronger for neighbours than non-neighbours (although not significant) and that effects on trust in coethnics are strongest when heterogeneity is aggregated to larger areas (although not significant).
